# Myocardial Scar Detection by Standard CT Coronary Angiography

**DOI:** 10.14740/cr341w

**Published:** 2014-07-20

**Authors:** Anand Jeevarethinam, Shreenidhi Venuraju, Vishal Shahil Mehta, Satvir Atwal, Usha Raval, Roby Rakhit, Joseph Davar, Avijit Lahiri

**Affiliations:** a Clinical Imaging and Research Centre, Wellington Hospital, Wellington Place, St. Johns Wood, London NW8 9LE, UK; b Institute of Cardiovascular Science, University College London, London, UK; c Royal Free and University College Medical School, UK; d Royal Free Hospital, London, UK; e Imperial College, London, UK; f Middlesex University, London, UK

**Keywords:** Myocardial scar, CT coronary angiogram, Cardiac imaging

## Abstract

We have described a myocardial infarct scar identified by a standard dual source CT coronary angiography (CTCA). We were able to detect the scar during the routine coronary assessment without contrast late enhancement and without additional radiation exposure. It is therefore feasible to assess chronic scar using a standard CTCA technique.

## Introduction

CT coronary angiography (CTCA) has become a robust and accurate imaging modality for the non-invasive assessment of coronary vessels. It is now being widely used, particularly in the low to intermediate cardiovascular risk group for the diagnosis and assessment of coronary disease severity [1].

It has been postulated that the data obtained from multi-detector CT (MDCT) during CTCA can also be used to quantify myocardial scar and viability but only by using delayed myocardial contrast enhancement, which involves a second, albeit low radiation dose [2, 3].

Late gadolinium-enhanced cardiac magnetic resonance imaging (CMRI) is considered the gold standard for detection of myocardial scar and viability, and few pilot studies have reported favorable results when comparing CTCA with MRI [2, 3].

Delayed enhancement studies usually offer more accurate information about the infarct size but at the cost of additional radiation exposure and more contrast. Cury et al demonstrated that patients with recent myocardial infarction (MI) (including sub-endocardial infarcts) could be detected using a standard MDCT dataset based on the presence of a perfusion defect (hypo-attenuation) with a sensitivity and specificity of 94% and 97% [4].

## Case Report

**Figure 1:**
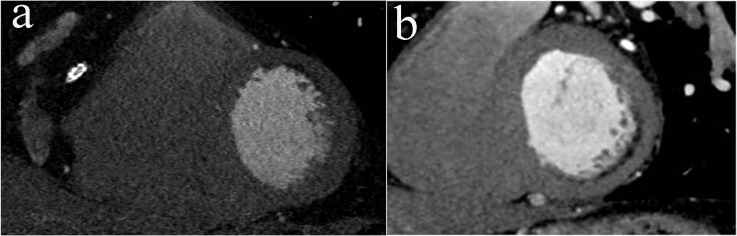
(a) Cardiac MDCT short-axis image shows antero-lateral scar consistent with previous infarction. (b) Cardiac MDCT short-axis image shows infero-lateral scar consistent with previous infarction.

We report the case of a 63-year-old gentleman presenting with chest pain who had previously undergone multi-vessel PCI in 2005. He underwent a CTCA (SOMATOM Definition Dual Source CT scanner, Siemens Medical Systems, Forchheim, Germany) in March 2013 for the evaluation of his coronary arteries as well assessment of his stents, which revealed an antero-apical, antero-lateral and infero-lateral myocardial scar and wall motion abnormalities in the same territories (Fig. 1). The stents were patent and 60% proximal LAD-calcified plaque was noted. The CT protocol included the following scan parameters: tube rotation time 330 ms, detector collimation 32 × 0.6 mm, pitch 0.2 to 0.3, tube voltage 120 kV and current 300 to 350 mA. Patients were scanned supine, in a craniocaudal direction, while maintaining an inspiratory breath hold (10 to 14 s). Contrast transit time was determined with a test bolus injection. For angiographic data acquisition, a 60- to 90-mL bolus of iomeperon was injected at 5.5 mL/s through a peripheral vein, followed by 40 mL of saline at the same rate.

**Figure 2:**
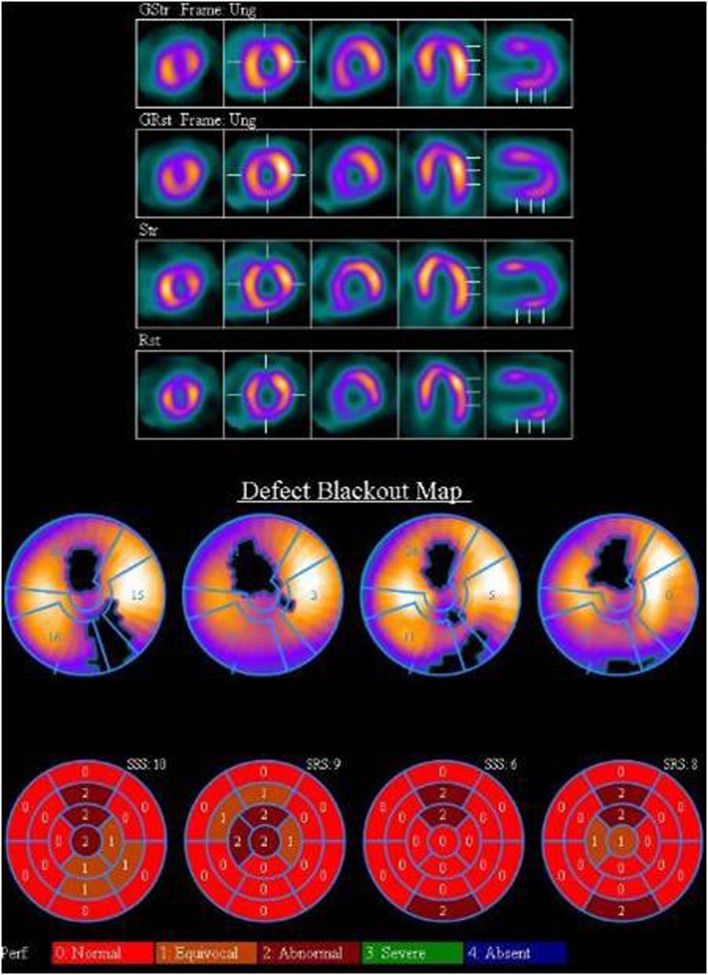
MPI SPECT shows antero-apical, anterior and infero-lateral fixed perfusion defects.

In view of proximal LAD lesion patient was referred for myocardial perfusion scan to assess ischemic burden. Stress (treadmill exercise + regadenoson) radionuclide myocardial perfusion imaging single photon emission CT (MPI SPECT) study with the 2 days protocol (^99m^Tc-sestamibi) was performed. SPECT slices and polar displays, illustrated on Figure 2, showed antero-apical, anterior and infero-lateral fixed perfusion defects in keeping with previous MI. Gated SPECT imaging showed anterior and infero-lateral wall motion abnormalities. It is worthy of note that MPI SPECT findings were consistent with CTCA findings.

## Discussion

Cardiac MRI offers high degree of spatial resolution and it has been used as a reference standard in detecting infract scars. But the presence of metallic devices precludes the use of cardiac MRI. The exponential rise in the use of CTCA as a diagnostic tool and considering similar physiological characteristics of iodinated contrast media and Gadolinium, there has been a sustained interest in utilizing it for the quantification of myocardial scar and to detect viability.

Gerber et al [5] showed attenuation patterns on CTCA enabling characterization of myocardial injury; early hypo-enhancement pattern reflecting acute MI causing micro-vascular obstruction and delayed hyper-enhancement reflecting chronic infarcts and extension of acute infarcts in contrast-enhanced MDCT. However, Nicol et al [6] emphasized that early hypo-enhancement may not be specific for myocardial injury unless there is concomitant wall motion abnormality. Stirrup et al [7] rightly pointed out that delayed wash-in of contrast during early hypo-attenuation could be under perfusion but not necessarily infarction.

Nieman et al [8] studied the ability of standard CTCA to elicit the difference between recent and longstanding MI on a group of patients who had recent MI < 7 days, previous MI > 12 months and no MI. He established recent MI patients had higher attenuation value (26+/-26 HU) than long standing MI (-13+/-37 HU) on 64-slice MDCT which implies recent and long-standing MIs could be differentiated by CTCA based on myocardial CT attenuation values.

In summary, we have described a myocardial infarct scar identified by a standard dual source CTCA. We were able to detect the scar during the routine coronary assessment without contrast late enhancement and without additional radiation exposure. It is therefore feasible to assess chronic scar using CTCA which could also be of potential clinical benefit in electro-physiology (EP) studies. Also it may be of prognostic importance in patients with previous silent infarction and in patients presenting with atypical chest pain in whom infarct size could influence the future management. However, we need to develop a technique to improve the accuracy of scar detection on the standard CTCA dataset without the need for further contrast and radiation exposure.
